# Baseline for the Northeast Atlantic (58–70°N) intertidal *Mytilus* species complex (*Mytilus* spp.)

**DOI:** 10.1002/ece3.70197

**Published:** 2024-08-23

**Authors:** Tore Strohmeier, Øivind Strand, Barbro Taraldset Haugland, Antonio Agüera

**Affiliations:** ^1^ Institute of Maine Research Bergen Norway

**Keywords:** abundance, baseline, bivalve, distribution, intertidal, method, monitoring

## Abstract

Mussels (*Mytilus* spp.) are abundant in the North Atlantic, sessile, and sensitive to environmental change, and suitable as sentinels of environment and climate change of costal ecosystems. We aimed to determine the baseline for the Northeast Atlantic (58–70°N) *Mytilus* species complex, and to show the present distribution to surveys conducted 60 years ago. Baseline was obtained by investigating a total of 509 stations in the intertidal zone, in four regions comprising the environmental gradient from head of fjord to coast, and distributed over the latitudinal gradient from 58 to 70°N. The baseline shows a range in continuous abundance of mussels from 12% to 36%, patchy abundance from 26% to 57% and no or very limited mussel abundance from 26% to 46% between the four regions. The presence of mussels in the southeast and west region was visualized to previous surveys conducted 60 years ago. The data points to similar past and present presence of mussels in both regions, yet past major mussel fields in the inner section of region southeast was not detected in this study. The baseline of *Mytilus* spp. in the Northeast Atlantic (58–70°N) is now available for future reference. The baseline, plotted to surveys conducted 60 years ago, points to awareness of the population situated in the southeast section of the investigated region. Continued monitoring and modeling are needed to clarify drivers of temporal and spatial variation in the mussel populations along the Northeastern Atlantic coast.

## Graphical abstract

The baseline of Mytilus spp. in the Northeast Atlantic (58–70°N) is now available for future reference. The baseline, showed to surveys conducted 60 years ago, points to awareness of the population situated in the southeast section of the investigated region.

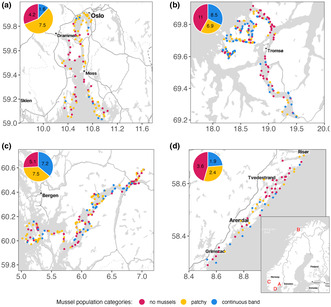

## 1 INTRODUCTION

Mussels (*Mytilus* spp.) are key species, ecosystem engineers and provide many ecosystem services in intertidal and shallow coastal ecosystems (Smaal et al., 2019). Mussels (*Mytilus* spp.) are abundant in the North Atlantic, sessile, and sensitive to environmental change, and suitable as sentinels of environment and climate change of costal ecosystems. During the last decades mussels have shown a poleward shift in the distribution along the Atlantic coast (Jones et al., 2010; Leopold et al., 2019) conceivably driven by elevated sea‐surface temperatures (Berge et al., 2005). Within Atlantic Europe there are increasing numbers of reports of declining mussel populations in coastal waters, which have been reviewed by Baden et al. (2021). Massive mussel die‐off was observed along the Netherlands (Capelle et al., 2021), in France (Charles et al., 2020), the Adriatic coast (Bracchetti et al., 2023) and decreased availability of wild seed in Galicia (Bracchetti et al., 2023; Padin et al., 2024). In Norway the Institute of Marine Research (IMR) has received an increasing number of notices from the public on observations of absence of local mussel populations during the last decade (Andersen et al., 2017). Based on a general concern for the disappearances and population changes of a key intertidal species, the IMR established a monitoring program for the mussel populations along the Norwegian coast.

Mussel distribution has been investigated in Norway approximately 60 years ago by Bøhle (1965) and Brattegard (1966). Bøhle (1965) surveyed the Oslofjord with the purpose of identifying “mussel fields” from the intertidal zone and to approximately 2 m depth (Bøhle pers. com.). Brattegard (1966) described the horizontal distribution of the fauna of rocky shores in the Hardangerfjord, based on field work conducted during 1955–63. We aim to compile, describe and to visualize the present and past distribution data of mussels where these studies overlap.

The Norwegian coastline, including the mainland, fjords and the islands is roughly 100,000 km long. Mussels are present along the entire coast (https://www.artsdatabanken.no/). Across the Trans‐Atlantic distribution, *Mytilus* species show interregional separation, with hybrid zones and mixed populations in the contact area (Wenne et al., 2020). The *Mytilus* species complex along the Norwegian coast comprises *M. edulis*, *M. trossulus*, *M. galloprovincialis* and their hybrids (Brooks & Farmen, 2013; Mathiesen et al., 2017; Wenne et al., 2020), yet their detailed geographical distribution along the Norwegian coast is not well known. Salinity and temperature are typically the main drivers for the distribution of the species on a large geographical scale (Gosling, 2021). Numerous abiotic (ice cover, substrate, turbidity, wave action, current speed, etc) and biotic factors (predators, disease, food availability, competitors, fouling organisms, etc) may influence population structure, abundance and local distribution patterns. Predation is often considered the single most important source for mortality and may influence local distribution pattern (Gosling, 2003).

The vast coastal area entails a method that can monitor environmental gradients from head of fjord to coast (i.e. up to several 100 km) and concurrently identify local changes in abundance. This called for an elementary and rapid categorization of mussel abundance and metadata (associated flora and fauna and habitat description), that would enable large spatial coverage.

In this letter we present the baseline for the North Atlantic intertidal mussel populations (58–70°N) and show the baseline with data from two separate surveys conducted 60 years ago. The objective of the monitoring program is to elucidate if the abundance of intertidal mussels is changing over time beyond the natural variation, i.e. beyond the inherent randomness in the ecosystems. Collection of additional metadata is included to describe the ecosystem and to assess potential cause(s) to change and will be presented when the monitoring has accrued enough data.

## 2 MATERIALS AND METHODS

**FIGURE 1:**
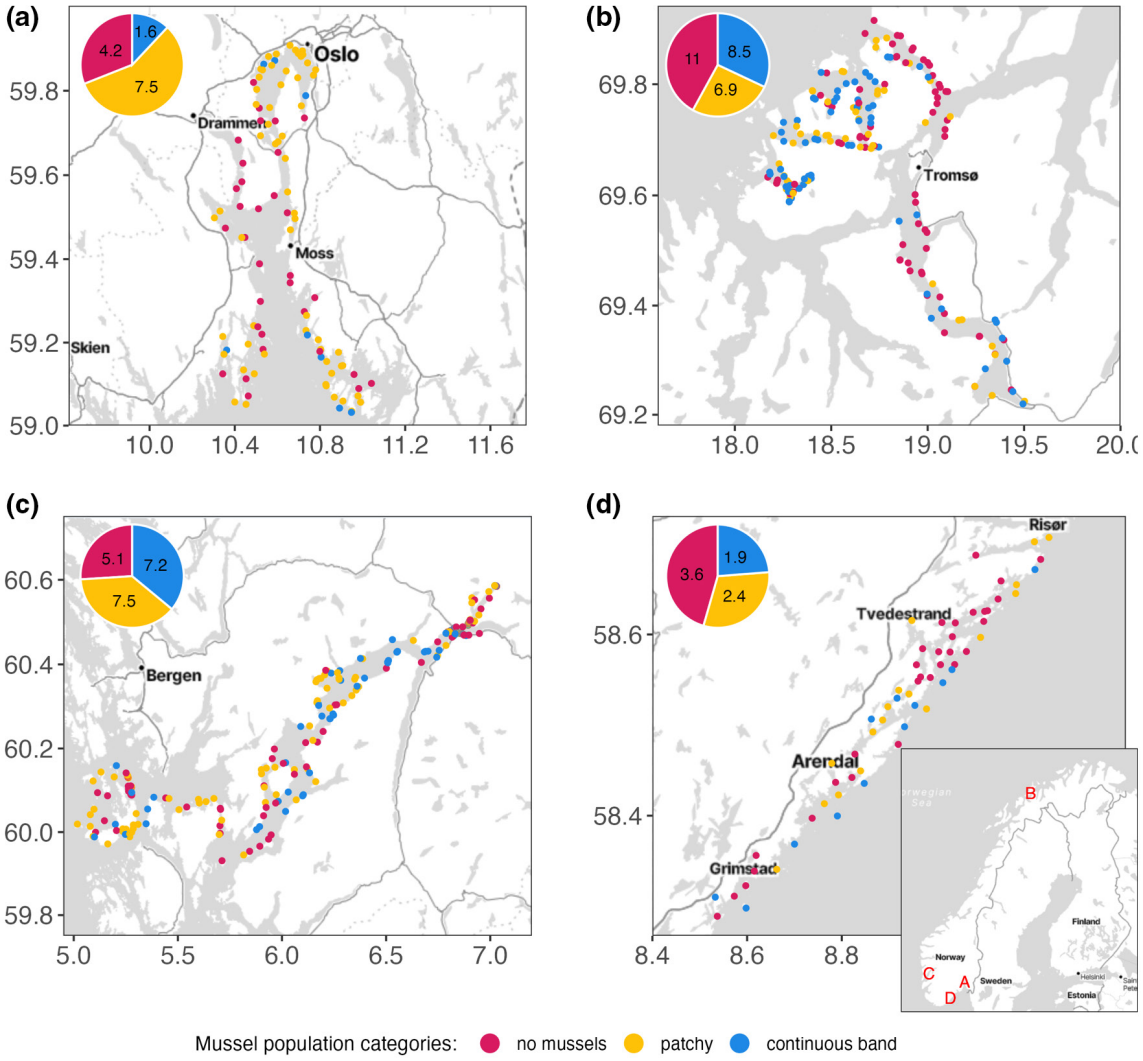
Monitored regions along the Norwegian coast (insert lower right) and baseline of mussel abundance (*Mytilus* spp.) in A = east, B = north, C = west and D = south in Norway during 2021 and 2022. Blue dots (i.e. line sections) indicate continuous abundance, yellow dots indicate patchy abundance and red dot indicate no or very limited abundance of mussels. Pie charts show the total distance (km) of the observed abundance category within each region. Note to panel C (region west): The relative presence of mussels detected in this study is compared to the findings of Brattegard (1966) east of 5.7°E (Figure A2), matching his subdivision of the fjord (i.e. fjord branches, inner fjord and intermediate fjord).

This baseline of intertidal mussel (*Mytilus* spp.) abundance comprises four investigated regions across latitudinal (58–70°N) and longitudinal (6–19°E) gradients along the North Atlantic coastline. Mussel abundance was surveyed over the environmental gradient from protected head of fjord to fully exposed coast. Based on the distribution of observations from the public on the absence of mussels (*Mytilus* spp.) (Andersen et al., 2017) we monitor four regions in Norway (east (A), north (B), west (C), south (D), Figure 1). As it is not possible to visually distinguish between the different species (or hybrids) in situ, this monitoring program reports the *Mytilus* species complex.

**FIGURE A1:**
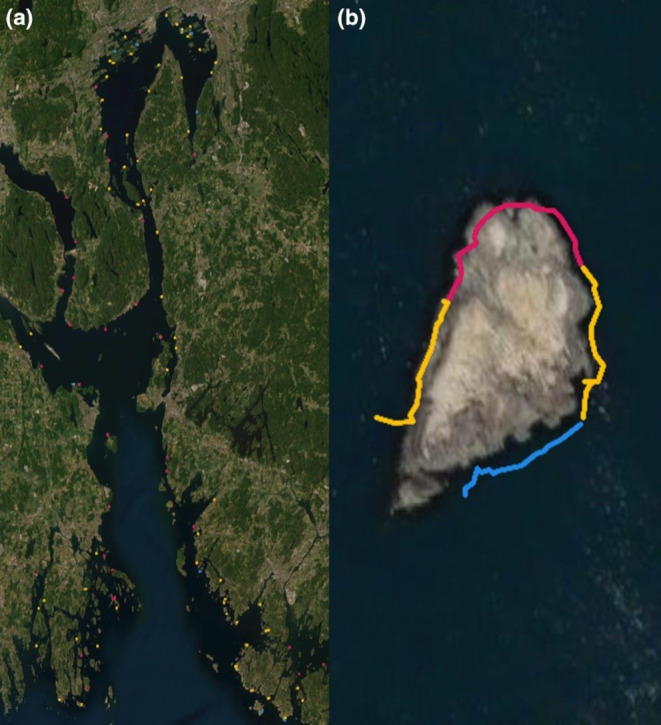
Screen display from the online application packages mussel population survey. Panel A: Surveyed station in region east in 2021 and 2022. Dots indicate line sections, green = continuous abundance, yellow = patchy distribution and red dot = no or very limited abundance of mussels (*Mytilus* spp.). Panel B: Magnification of an investigated line section (total length 174 m). Line color indicate mussel abundance as given for dots.

Stations within each region were obtained using randomized line sections. Selection of random line sections was done using Python v3 (Van Rossum & Drake, 1995), in short a high‐resolution polygon representing the coastline of the area, including islands, was divided in 100 m line sections, these sections are then numbered, and a random number generator was used to randomly select the needed number of line sections (50–100 per region in 2021 and 2022). Each station (line section) comprises 100 m of coastline, horizontally in the intertidal zone. An example of a sampled line section is given in Figure A1. Stations falling within restricted and protected areas (nature reserves, bird nesting sites, military area, etc) and danger‐ or temporally non accessible areas (busy harbors, wind and waves action, etc) were ignored. The main cause for ignoring stations were wind and wave action. This was compensated for by surveying exposed locations during the most favorable wind conditions during the campaign, yet sites directly exposed to open ocean have sometimes been ignored. Few stations (less than 10%) were ignored by other causes. Surveying one region requires approximately 120 h of fieldwork (three persons × 10 h × 4 days).

The abundance of mussels was determined by the degree of horizontal coverage along the generated line sections of the intertidal zone and as deep as the water visibility allowed for. Observations were acquired either by snorkeling or walking along the intertidal zone. Snorkeling has been preferred for the three southern regions where the tidal range is typically <1 m. Snorkeling has also been preferred for habitats with abundant vegetation, boulders, cracks, steep inclination or else where it is difficult to walk in the intertidal zone. The observer is fitted with a GPS that logs geo‐referenced positions at 0.2 Hz. The observer reports mussel abundance in three categories (see criteria below), assess the presence of age classes, dominant substrate (rock, boulder, pebbles, sand or mud) and a species list of dominant flora and the most common mussel predators. Age classes were determined visually based on the absence or presence of annual (winter) concentric growth rings; age class 0+ showed no annual concentric growth ring, age class 1+ showed one annual concentric growth ring and age class 2++ showed two or more annual concentric growth rings. Two online application packages have been developed to log (Agüera, 2024b) and explore (Agüera, 2024a) the data.

**FIGURE 2:**
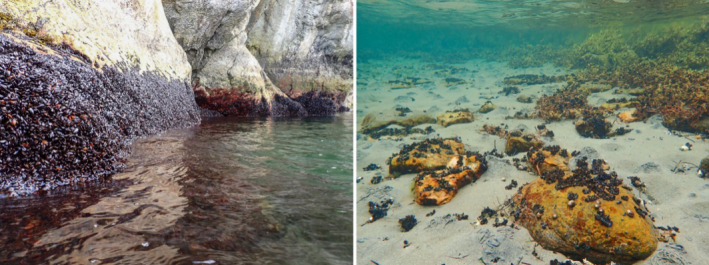
Pictures of mussel (*Mytilus* spp.) from two different habitats representing abundance category 1 (continuous belt, left) and category 2 (patchy, right).

The abundance of mussels is determined by the degree of horizontal coverage, using three categories. An example of category 1 and 2 is given in Figure 2.

**Category 1: Continuous abundance**: Continuous horizontal coverage of mussels in the habitat. Mussels are occupying 100% of an imaginary horizontal line extending >2 m along the station. The imaginary horizontal line implies that mussels must not be situated next to each other as overlapping vertical spaced (patches of) mussels may fulfill the criteria. The width (i.e. the vertical height or “thickness”) of the horizontal line is not considered. Up to a 2 m break in continuous cover is accepted before a shift in category is reported.

**Category 2: Patchy abundance:** Patchy horizontal coverage of mussels in the habitat. Patchy abundance of mussels includes continuous abundance of mussels extending <2 m in horizontal direction, groups of mussels and individual mussels. Patchy abundance allows a maximum horizontal distance between observations of mussels of 5 m.

**Category 3: Non or very limited abundance of mussels**: Singular or groups comprising few mussels may appear on distances exceeding 5 m, yet mussels are normally not observed.

## 3 RESULTS AND DISCUSSION

The baseline comprises 509 investigated stations obtained from the four regions sampled during 2021 and 2022 (Figure 1). In average, 16‐line sections were surveyed per day of field work. The mean length of all sampled stations was 133 m and varied less than ±5 m between regions (range 128–137 m). The total length of the coastline investigated was 60 km. The number of stations in West was 155, in East was 100, in North was 194 and South was 60. South was only monitored in 2022.

The baseline of abundance of mussels (*Mytilus* spp.) within the different regions is in Figure 1. We did not see a common clear pattern in mussel abundance over the environmental gradient from head of fjord to exposed coast. Continuous abundance of mussels (category 1) was present along 12%–36% of the total investigated coastline, where the western region had the highest percentage occurrence and the eastern region the lowest. The range in percentage occurrence of patchy abundance of mussels (category 2) spans from 26%–57% between regions, where the eastern region shows the highest and the north region the lowest. No or very limited mussel abundance (category 3) ranged 26%–46% in occurrence, with the western region showing the lowest and the southern region having most observation of category 3. Overall, we detected more continuous and patchy distribution than non or very limited mussels in all regions, across the latitudinal (58–70°N) and longitudinal (6–19°E) gradients. The baseline of *Mytilus* spp., along four regions of the Norwegian coast, is now available for future reference and will be decisive for our future understanding of the temporal and spatial variation in these mussel populations.

Mussel distribution was investigated in the southeastern and western regions approximately 60 years ago by Bøhle (1965) and Brattegard (1966). Bøhle (1965) surveyed the Oslofjord, corresponding with our southeastern region, with the purpose of identifying “mussel fields” from the intertidal zone and to 2 m depth. Bøhle's sampling sites and mussel fields are visualized in his Figure 2, which corresponds to our Figure 1 (panel A). Bøhle (1965) reports the highest presence and abundance of mussels in the inner part of the Oslofjord (north from Drøbak), the southwestern part of the region (Tønsberg and Nøtterøy) as having “good mussel fields” and the mid part of the region (Hurumlandet to Holmestrandsfjorden) as having “good abundance” of mussel. His observations most often resemble our findings of presence for similar situated sampling stations, however major “mussel fields” in the inner Oslofjord (Nesodden and Sætre) and mussel abundance in the effluent area of the Drammen River was not detected in this survey. This points to awareness of these populations and continued monitoring should clarify if the populations are changing.

**FIGURE A2:**
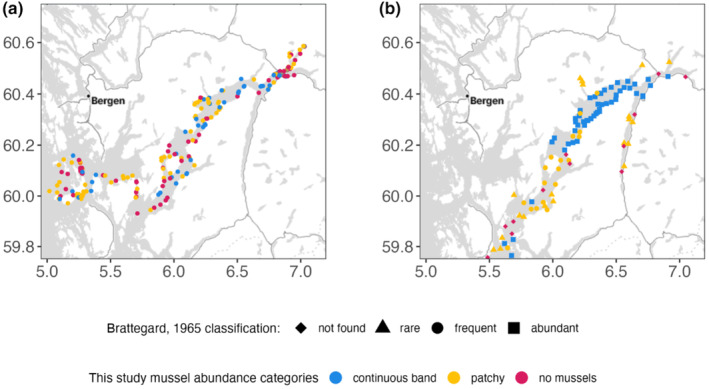
The relative presence of mussels reported by Brattegard, 1966 east of 5.7°E. Blue markers indicate abundant, yellow markers indicate patchy distribution and red markers indicate not found.

Brattegard (1966) described the horizontal distribution of the fauna of rocky shores in the Hardangerfjord, based on field work conducted during 1955–63. His study site overlaps with fjord branches, inner fjord, and intermediate fjord in the western region of our study (see Figure A2). These maps of past and present distribution show minor changes in presence but indicates that mussels currently are more present in the fjord branches and less present in the inner part and the intermediate section of the fjord. Brattegard's study was designed to describe the fauna on rocky shores, and protruding headlands and rocky substrata were selected for the investigated stations. This survey should include all substrates and habitats in the region, also habitats where mussels may not thrive, such as sandy or pebble beaches, freshwater outlets, etc. It is therefore to be expected that our approach will detect lower presence. Given the minor differences in presence (including a possible change in genetic composition) between the past and present survey and the different methodology employed, it appears inconclusive if the mussel presence in the Hardangerfjord has changed over the last 60 years.

The method used in this study has been applied in many different habitats and should be applicable for other regions. We collect data from random obtained stations which allows for future statistical comparisons within and between regions and the use of random sites should also ensure that most of the present habitats will be surveyed. The method is efficient for most habitats, especially when mussels are abundant (i.e. for category 1 and 2 sites), yet more time consuming when mussels are sparse or absent in habitats comprising dense vegetation, boulders or at gently sloping seabed. The method and app also allow for reporting substrate, flora and fauna, merging pictures to the line section and to report age classes. However, the data collected is limited in the use to assess the stability of mussel bed based on the lack of a quantification of the age structure of the site/population. It may also be difficult to discern the number of growth rings in situ, especially from mussels residing in the upper intertidal zone. This has limited the age classification to 2 years old mussels in this baseline study.

The dataset presented here represents a baseline and a starting effort to compile knowledge on the habitat and biology and distribution of the *Mytilus* complex along the Northeastern Atlantic coast. The baseline and metadata are collected to assess the role of ecosystem interactions (predation, competition) and habitat characteristics (substrate, vegetation) in the distribution biology of the *Mytilus* complex. Our goal is to generate spatial distribution models and ecological niche time series for the mussels from the different regions by combining the baseline data with environmental variables generated from oceanographic models (NorKyst800, (Asplin et al., 2020)) and ecosystem models (Norwecom.e2e, (Skogen et al., 1995)). These models should aid to assess the role of changes in environmental forcing on the distribution of mussels in the studied areas.

## 4 SIGNIFICANCE STATEMENT

Mussels (*Mytilus* spp.) are abundant in the North Atlantic, sessile, and sensitive to environmental change, and suitable as sentinels of environment and climate change of costal ecosystems. During the last decades mussels have shown a poleward shift in the distribution along the Atlantic coast conceivably driven by elevated sea‐surface temperatures. We have determined the baseline for mussels in the Northeast Atlantic, representing a geographical scale and habitat diversity previously not surveyed. The baseline has been visualized with two surveys conducted 60 years ago, and it points to awareness of the population situated in the southeast section of the investigated region.

## AUTHOR CONTRIBUTIONS

**Tore Strohmeier:** Conceptualization (lead); data curation (lead); formal analysis (equal); funding acquisition (lead); investigation (lead); methodology (lead); project administration (lead); resources (equal); software (supporting); visualization (supporting); writing – original draft (lead); writing – review and editing (lead). **Øivind Strand:** Conceptualization (supporting); data curation (supporting); formal analysis (supporting); investigation (equal); methodology (equal); project administration (supporting); software (supporting); visualization (supporting); writing – original draft (supporting); writing – review and editing (supporting). **Barbro Taraldset Haugland:** Conceptualization (supporting); data curation (supporting); formal analysis (supporting); investigation (supporting); methodology (supporting); software (supporting); visualization (supporting); writing – original draft (supporting); writing – review and editing (supporting). **Antonio Agüera:** Conceptualization (supporting); data curation (supporting); formal analysis (supporting); investigation (supporting); methodology (supporting); software (lead); visualization (lead); writing – original draft (supporting); writing – review and editing (supporting).

## CONFLICT OF INTEREST STATEMENT

The authors have no conflict of interest to declare.

## Data Availability

Data displayed in this study are available at https://zenodo.org/doi/10.5281/zenodo.12818271 and can be explored in detail here https://shellfish.shinyapps.io/mussel_distribution_explorer/. The code for the tool to collect the data is available https://zenodo.org/doi/10.5281/zenodo.12779387 with a running example that can be explored here https://www.shinyapps.io/admin/#/application/10804254.
